# Tracking of autologous adipose tissue-derived mesenchymal stromal cells with *in vivo* magnetic resonance imaging and histology after intralesional treatment of artificial equine tendon lesions - a pilot study

**DOI:** 10.1186/s13287-016-0281-8

**Published:** 2016-02-01

**Authors:** Florian Geburek, Kathrin Mundle, Sabine Conrad, Maren Hellige, Ulrich Walliser, Hans T. M. van Schie, René van Weeren, Thomas Skutella, Peter M. Stadler

**Affiliations:** Clinic for Horses, University of Veterinary Medicine Hannover, Foundation, Bünteweg 9, 30559 Hannover, Germany; Pferdeklink Kirchheim, Nürtinger Straße 200, 73230 Kirchheim unter Teck, Germany; P.O. Box 1243, 72072 Tübingen, Germany; Department of Equine Sciences, Faculty of Veterinary Medicine, Utrecht University, Yalelaan 112, 3584 CM Utrecht, The Netherlands; Institute for Anatomy and Cell Biology, University of Heidelberg, Im Neuenheimer Feld 307, 69120 Heidelberg, Germany

**Keywords:** Horse, Superficial digital flexor tendon, Superparamagnetic iron oxide particles, SPIO, Prussian blue staining, Green fluorescent protein

## Abstract

**Background:**

Adipose tissue-derived mesenchymal stromal cells (AT-MSCs) are frequently used to treat equine tendinopathies. Up to now, knowledge about the fate of autologous AT-MSCs after intralesional injection into equine superficial digital flexor tendons (SDFTs) is very limited. The purpose of this study was to monitor the presence of intralesionally injected autologous AT-MSCs labelled with superparamagnetic iron oxide (SPIO) nanoparticles and green fluorescent protein (GFP) over a staggered period of 3 to 9 weeks with standing magnetic resonance imaging (MRI) and histology.

**Methods:**

Four adult warmblood horses received a unilateral injection of 10 × 10^6^ autologous AT-MSCs into surgically created front-limb SDFT lesions. Administered AT-MSCs expressed lentivirally transduced reporter genes for GFP and were co-labelled with SPIO particles in three horses. The presence of AT-MSCs in SDFTs was evaluated by repeated examinations with standing low-field MRI in two horses and post-mortem in all horses with Prussian blue staining, fluorescence microscopy and with immunofluorescence and immunohistochemistry using anti-GFP antibodies at 3, 5, 7 and 9 weeks after treatment.

**Results:**

AT-MSCs labelled with SPIO particles were detectable in treated SDFTs during each MRI in T2*- and T1-weighted sequences until the end of the observation period. Post-mortem examinations revealed that all treated tendons contained high numbers of SPIO- and GFP-labelled cells.

**Conclusions:**

Standing low-field MRI has the potential to track SPIO-labelled AT-MSCs successfully. Histology, fluorescence microscopy, immunofluorescence and immunohistochemistry are efficient tools to detect labelled AT-MSCs after intralesional injection into surgically created equine SDFT lesions. Intralesional injection of 10 × 10^6^ AT-MSCs leads to the presence of high numbers of AT-MSCs in and around surgically created tendon lesions for up to 9 weeks. Integration of injected AT-MSCs into healing tendon tissue is an essential pathway after intralesional administration. Injection techniques have to be chosen deliberately to avoid reflux of the cell substrate injected. *In vivo* low-field MRI may be used as a non-invasive tool to monitor homing and engraftment of AT-MSCs in horses with tendinopathy of the SDFT.

## Background

Injury of the superficial digital flexor tendon (SDFT) is common, especially in thoroughbred racehorses [1]. Therapy with adipose-derived nucleated cells and adipose tissue-derived mesenchymal stromal cells (AT-MSCs) has shown promising results when treating both experimental tendonitis [2–5] and naturally occurring tendon and ligament injuries in horses [6, 7]. To further investigate the potential regenerative, immunomodulatory and inflammatory modulating effect of MSC therapy, knowledge of the distribution, kinetics and engraftment of the injected cells in the target tissue is of utmost interest [8]. Bone marrow-derived MSCs (BM-MSCs) and embryonic-like stem cells (ESCs) have been traced after labelling with green fluorescent protein (GFP) and were detectable in the treated tendon for up to 90 days [9, 10]. Whereas estimated BM-MSC survival was less than 5 % after 10 days, ESC numbers were at a constant level for 90 days [10]. Compared with MSCs from other sources, AT-MSCs show expression of extracellular matrix proteins with the highest collagen 1A2-to-collagen 3A1 ratios. Moreover, AT-MSCs display the second highest expression of the tendon markers tenascin-C and scleraxis [11], suggesting that AT-MSCs may be of special benefit in the treatment of equine tendinopathy. However, knowledge about the fate of autologous AT-MSCs after intralesional injection into equine SDFTs is marginal. As recently revealed, AT-MSCs labelled with nanocrystals were detectable in peripheral blood for 72 h and 7 days after implantation as well as in SDFT tissue 7 days after intralesional injection in an equine collagenase tendonitis model [12].

Labelling with superparamagnetic iron oxide (SPIO) nanoparticles is a technique to track MSCs assisted by magnetic resonance imaging (MRI) because of the strength of the signal change per unit of metal provided, particularly in T2*-weighted images [13]. By means of this technique, cells can be monitored non-invasively *in vivo* and at post-mortem histology by Prussian blue staining [14]. In contrast, GFP-based labelling techniques are dependent on tissue biopsies or even larger specimen, thus making euthanasia of the treated animal necessary [10]. *In vitro* studies have shown that controlled labelling of MSCs with SPIO nanoparticles neither caused death of rabbit BM-MSCs nor inhibited their proliferation [15]. A recent equine study has provided evidence that viability did not differ between SPIO-labelled and unlabelled BM-MSCs and umbilical cord blood MSCs. However, doubling time increased in SPIO-labelled MSCs compared with unlabelled cells [16]. In a rodent study, SPIO nanoparticles could be tracked *in vivo* for up to 4 weeks after subcutaneous implantation [17]. At the same time, in a different rodent study investigating the presence of SPIO-labelled BM-MSCs at a tendon-to-bone interface for up to 7 days, a reliable tracing of labelled cells was impossible and this was due to the similar signal intensity of cells and tendon tissue on T2-weighted MRI images [18]. As recently pointed out in an equine cadaver study, SPIO-labelled BM-MSCs are detectable immediately after intralesional SDFT injection by using 1.5-Tesla MRI [16].

The present pilot study aimed at testing whether standing low-field MRI has the potential to monitor the fate of intralesionally injected AT-MSCs labelled with SPIO particles *in vivo* and at monitoring the presence of AT-MSCs that were co-labelled with GFP histologically for up to 9 weeks in a surgical model of equine tendinopathy.

## Methods

Four warmblood horses (two stallions, one mare and one gelding) between 1 and 4 years old were objects of this study. Pre-existing tendon injury was excluded by clinical examination, B-mode ultrasonography and ultrasonographic tissue characterization (UTC) (UTC 2009; UTC Imaging, Stein, The Netherlands). The study was approved by the animal welfare officer of the University of Veterinary Medicine Hannover, Foundation, Germany, and the ethics committee of the responsible German federal state authority in accordance with the German Animal Welfare Law (Lower Saxony State Office for Consumer Protection and Food Safety, Az. 33.9-42502-04-08/1622).

### Collection of subcutaneous fat, AT-MSC isolation, and culture

After sedation of the horses, approximately 1–2 g of subcutaneous fat was harvested from the left coccygeal region at the base of the tail 8 or 9 days prior to surgical creation of standardized SDFT lesions. AT-MSCs were isolated and cultured as described elsewhere [4]. They were defined by the presence of markers CD44, CD90, CD105 and CD117 and the absence of CD34 and CD45.

### Labelling with lentiviral plasmid and superparamagnetic iron oxide particles

**Fig. 1:**
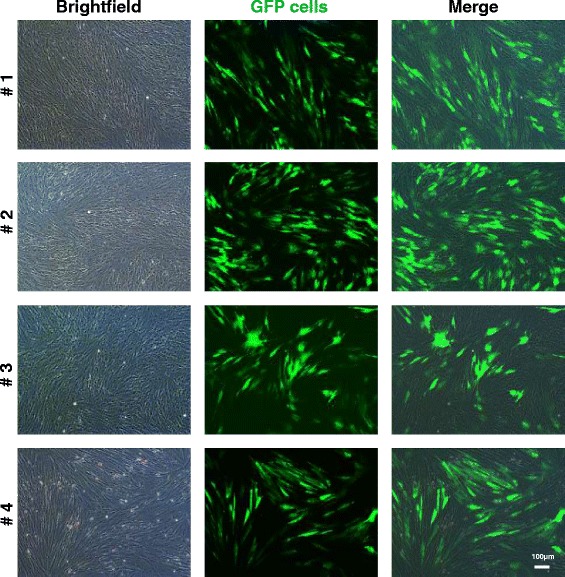
Photomicrographs of cultured adipose-derived mesenchymal stromal cells from horses 1 to 4 after transfection with lentiviral green fluorescent protein (GFP) construct. Bright field (*left*), fluorescence (*middle*) and merged (*right*) images are shown. Approximately 70–90 % of the cells were positive for GFP in their cytoplasm during passage 1. Magnification: 10×. Scale bar: 100 μm

**Fig. 2:**
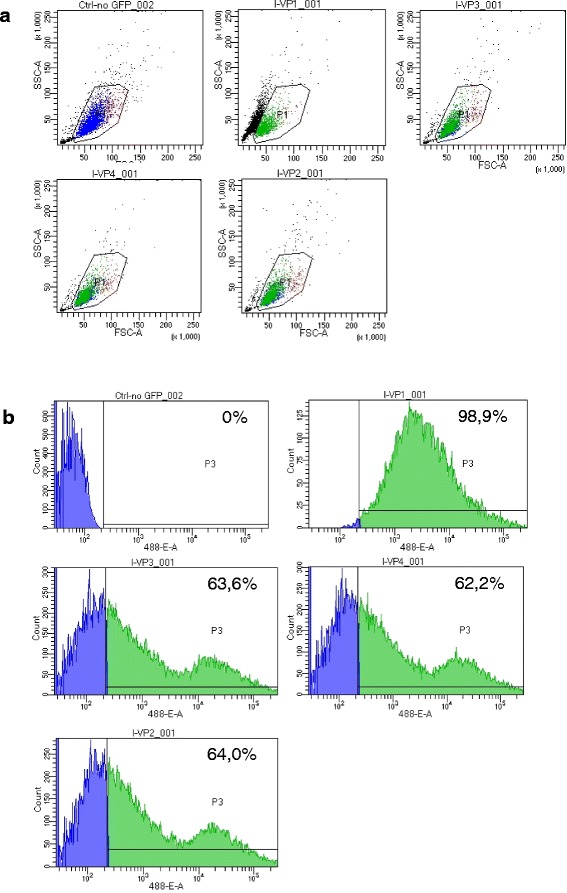
Flow cytometric analysis of cultured AT-MSCs from horses 1 to 4 after transfection with lentiviral green fluorescent protein (GFP) construct. From left to right are (**a**) scatter plots and (**b**) histograms, including percentages showing the distribution of GFP-expressing cells in a non-expressing control sample (ctrl-no GFP) and in samples from horse 2 (I-VP1; passage 0) and horses 1, 3 and 4 (I-VP3, I-VP4 and I-VP2; passage 2) after a single analysis. Green indicates GFP-labelled AT-MSCs, and blue indicates non-labelled AT-MSCs. *AT-MSC* adipose tissue-derived mesenchymal stromal cell, *FSC-A* forward scatter A, *GFP* green fluorescent protein, *SSC-A* side scatter A

After the addition of 10 μg/ml polybrene, the cultured AT-MSCs of all horses were incubated with lentivirus particles with a copGFP (hUbiC Promoter) for 48 h. The efficacy of transfection was controlled by fluorescence microscopy during passage 1 (Fig. 1) and by flow cytometric analysis (Fig. 2) which was performed on a BD FACSCanto™ II with BD FACSDiva™ 8.0.1 software (BD Biosciences, Franklin Lakes, NJ, USA). For excitation, the 488-nm laser line was used. Debris, dead or damaged cells as well as cell aggregates were excluded from further analysis according to forward scatter (FSC) and side scatter (SSC) properties (gate P1 and P2). For the detection of GFP fluorescence intensity, a combination of a 502 low-pass (LP) and 530/30 band-pass (BP) filter in front of the photomultiplier tube (PMT) detector (488-E) was employed. Detection sensitivity was set with a non-expressing control sample. The GFP-positive gate (P3) was set on the basis of the negative control population to include 0.0 % positive events. The mean efficacy of transfection was estimated to be 99 % after passage 0 (horse 2) and 63 %, 62 %, and 64 % after passage 2 (horses 1, 3 and 4) as determined by three analyses, respectively.

Additionally, AT-MSCs from horses 1, 2 and 4 were labelled by incubation with 45- to 60-nm SPIO particles coated with carboxydextran (Resovist™, SHU 555A; Schering AG, Berlin, Germany) in culture medium for 48 h at an SPIO concentration of 60 μg/ml Fe. Following the incubation, AT-MSCs were rinsed thrice with phosphate-buffered saline (PBS) solution. After harvest of AT-MSCs from passage 0, the total number of cells was counted and the cell viability evaluated, followed by sample preparation of 10 × 10^6^ AT-MSCs on ice for transplantation. Cells not needed for transplantation were cryopreserved for further experiments.

### Creation of superficial digital flexor tendon lesions

Lesions were created in the SDFTs of both front limbs under general anaesthesia by using a technique previously described [19–21]. A short longitudinal incision was made axially at the palmar aspect of the metacarpus just proximal to the digital sheath. A conical obturator (Ø 3.5 mm) from an arthroscope was introduced through a stab incision into the center of the SDFT and advanced proximally for 7–8 cm while carefully avoiding penetration of the epitenon. The obturator was replaced by an arthroscopic burr (Small Joint Round Burr ø 3.5 mm, 28204 FB; Karl Storz GmbH & Co. KG, Tuttlingen, Germany), which was activated and slowly withdrawn within approximately 20 s. The incisions were closed in a simple interrupted pattern. The horses received meloxicam (0.6 mg/kg body weight (bwt) [orally]) the day of surgery and for two consecutive days.

### Injection of labelled AT-MSCs

The tendon lesions were treated unilaterally with AT-MSCs at 1 week (horses 2 and 3) and at 3 weeks (horses 1 and 4) after surgery. Three horses (1, 2 and 4) received AT-MSCs labelled with SPIO particles. The horses were sedated by using detomidine hydrochlorid (0.02 mg/kg bwt [IV]) and butorphanol (0.04 mg/kg bwt [IV]). Additionally the medial and lateral palmar nerves were anaesthetized with 2 ml of 2 % mepivacaine hydrochlorid 2 cm distal to the carpometacarpal joint. Approximately 10 × 10^6^ AT-MSCs suspended in 2 ml 0.9 % saline (total volume 3 ml) and divided into aliquots of 1.5 ml were injected into the SDFT lesions from lateral under ultrasonographic guidance by using a 23-G, 1-inch needle. Entrance of the needle was 3 and 5 cm proximal to the surgical scar. Following the implantation of the cells, the limbs were dressed with half-limb bandages for 16 days. The contralateral SDFT was treated with 3 ml of saline in the same manner.

### Standing low-field magnetic resonance imaging

In two of the horses treated with SPIO-labelled AT-MSCs (2 and 4), sequential standing low-field MRI of the metacarpal regions was performed by using a clinical 0.27-Tesla open magnet (Hallmarq Veterinary Imaging, Guildford, Surrey, UK) and a fetlock radiofrequency coil. Front-limb SDFTs were scanned 6 and 33 days after the intralesional treatment in horse 2 and after 2, 18, 47 and 62 days in horse 4.

Initially the horses were sedated with romifidine (0.06 mg/kg bwt [IV] and butorphanol (0.01 mg/kg bwt [IV]). The sedation was prolonged to effect with detomidine (0.01 mg/kg bwt [IV]). After positioning of the metacarpal regions in the isocenter of the magnet, the following MRI sequences and variables were applied: transverse gradient-echo T1-weighted, TR (relaxation time): 50 ms, TE (echo time): 8.0 ms, FA (flip angle): 50°, matrix size: 170 × 130, number of excitations: 1; transverse gradient-echo T2*-weighted, FA: 25°, matrix size: 170 × 126; number of excitations: 1; transverse STIR, TR: 2260 ms, TE: 22.0 ms, FA: 90°, matrix size: 168 × 168, number of excitations: 8. The field-of-view angle was 170 × 170 mm, the slide width was 5.0 mm and the gap width was 6.0 mm.

### Follow-up

Following the creation of the lesions, the horses received box rest for 3 weeks. Thereafter they were exercised in accordance with a protocol adapted from Bosch et al. (2010) [21] until euthanasia: From week 4 to 6, they were walked for 10 min once a day on a treadmill. During week 7 to 9, walking was extended to 20 min once daily. After sedation with xylazine (0.4 mg/kg bwt [IV]) and levomethadone (0.05 mg/kg bwt [IV]) and induction of general anaesthesia with ketamine (3 mg/kg bwt [IV]) and midazolam (0.02 mg/kg bwt [IV]), horses were euthanized by using an overdose of pentobarbital (80 mg/kg bwt [IV]) at 3, 5, 7 and 9 weeks after injection of the cells, respectively.

### Fluorescence microscopy and Prussian blue staining

The SDFTs were dissected immediately after death, and a tendon segment including the lesion was harvested from between 4 and 5 cm proximal to the surgical scar. These specimens were stored for 96 h in 4 % paraformaldehyde followed by 96 h in PBS. Thereafter they were snap-frozen in Tissue-Tek (Sakura, Alphen aan den Rijn, The Netherlands) at −180 °C. Transverse sections with a thickness of 10 μm were cut with a cryostat and distended on histology slides. Sections were fixed with 4 % paraformaldehyde for 10 min, washed with PBS and stored at −80 °C after drying until required. Slices were examined under a fluorescence microscope (20× magnification). A set of frozen sections was stained with Prussian blue as described elsewhere (immunohistochemistry world) and examined with a light microscope (20× magnification).

### Immunohistochemistry and immunofluorescence

The cell culture and frozen tissue sections were analysed immunohistochemically and with immunofluorescence to detect the GFP antigen. Incubations with the primary polyclonal antibody rabbit-Anti-CopGFP (Evrogen AB 513; Evrogen, Moscow, Russia) diluted at 1:1000 were carried out overnight at 4 °C. After incubation with the primary antibody, one set of sections was incubated with a biotylinated swine-anti-rabbit secondary antibody (E0431; Dako, Hamburg, Germany) at a dilution of 1:400 for 60 min at room temperature. Thereafter, the peroxidase streptavidin-biotin-complex (ABC) detection system (Vectastain Elite ABC Kit; Vector Laboratories Inc., Burlingame, CA, USA) was applied and slices were exposed to diaminobenzidine tetrachloride (DAB) substrate (Sigma-Aldrich, St. Louis, MO, USA) in accordance with the instructions of the manufacturer. A second set of sections was incubated with a secondary goat-anti-rabbit antibody Alexa 546 (Molecular Probes) at a dilution of 1:1000. Finally, the sections were counterstained with 4′,6-diamidino-2-phenylindole (DAPI) (D9542; Sigma-Aldrich) diluted at 1:2000. Cop-GFP transfected control cells functioned as positive controls. In the negative control sections, the primary antibody was omitted. During microscopy of all slices, the number of cells with a positive signal was estimated subjectively (low/moderate/high numbers).

## Results

### Creation of lesions

Horses 2 (left front) and 4 (both, right front > left front) developed a cellulitis. This was accompanied by the development of lameness, which started at 1 day after surgery and persisted for 5 days in horse 2. In horse 4, lameness started at 6 days after lesion creation and lasted for 2 days. Both horses received cefquinome for 7 days (1 mg/kg bwt [intramuscularly]).

### Histology of AT-MSCs in cell culture

**Fig. 3:**
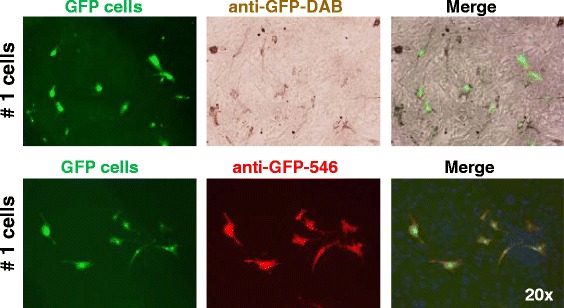
Photomicrographs of GFP-labelled equine AT-MSCs from horse 1 in cell culture. In the upper row, GFP-fluorescence, anti-GFP immunohistochemistry (DAB) and merged images are shown. In the lower row, GFP-fluorescence, anti-GFP immunofluorescence (Alexa 546) and merged images of AT-MSCs (passage 6) are shown. Magnification: 20×. *AT-MSC* adipose tissue-derived mesenchymal stromal cell, *DAB* diaminobenzidine tetrachloride, *DAPI* 4′,6-diamidino-2-phenylindole, *GFP* green fluorescent protein

Labelled AT-MSCs could be successfully detected in cell culture with fluorescence microscopy (Figs. 1, 3) and with immunohistochemistry and immunofluorescence by using DAB and Alexa 546, respectively (Fig. 3).

### Histological tracking of GFP-labelled AT-MSCs

**Fig. 4:**
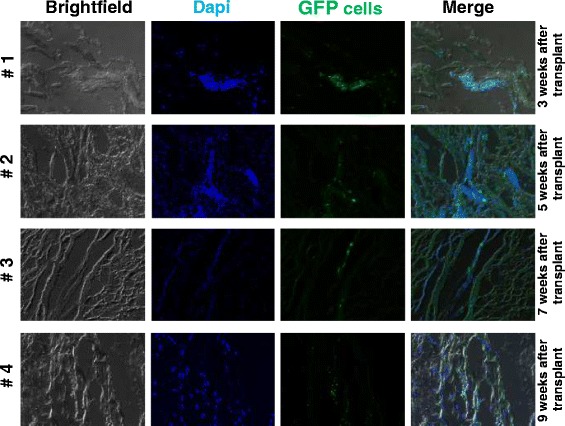
Photomicrographs of superficial digital flexor tendon lesions 3, 5, 7 and 9 weeks after transplantation of GFP-labelled autologous adipose tissue-derived mesenchymal stromal cells. Bright field, DAPI nuclear stain and GFP-fluorescence and merged images taken from horses 1–4 are shown. Transplanted cells were positive in the cytoplasm for GFP. Magnification: 20×. *DAPI* 4′,6-diamidino-2-phenylindole, *GFP* green fluorescent protein

**Fig. 5:**
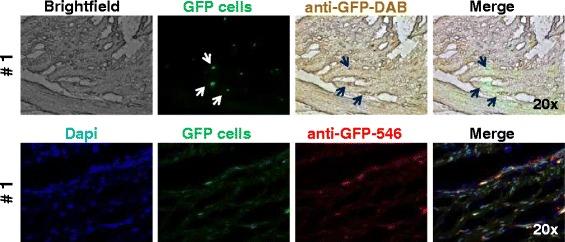
Photomicrographs of a superficial digital flexor tendon lesion 3 weeks after transplantation of GFP-labelled autologous adipose tissue-derived mesenchymal stromal cells. In the upper row, bright field, GFP-fluorescence, anti-GFP immunohistochemistry (DAB) and merged (*arrows*: positive cells) images are shown. In the lower row, DAPI nuclear stain, GFP-fluorescence, anti-GFP immunofluorescence (Alexa 546) and merged images (horse 1) are shown. Approximately 50 % of the cells were positive for GFP in their cytoplasm. Magnification: 20×. *DAB* diaminobenzidine tetrachloride, *DAPI* 4′,6-diamidino-2-phenylindole, *GFP* green fluorescent protein

**Fig. 6:**
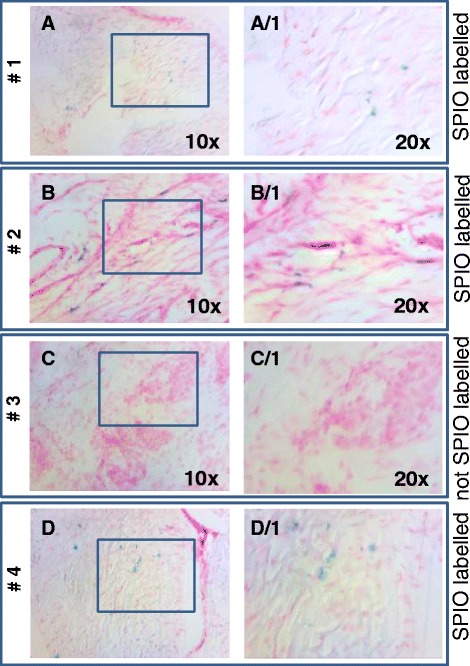
Photomicrographs of surgically created superficial digital flexor tendon lesions treated with autologous AT-MSCs and stained with Prussian blue. **a**, **b**, **d** Lesions treated with SPIO-labelled AT-MSCs (horses 1, 2 and 4). **c** Lesion treated with non-SPIO-labelled AT-MSCs (horse 3). Magnifications: 10× (a-d) and 20× (A/1-D/1). *AT-MSC* adipose tissue-derived mesenchymal stromal cell, *SPIO* superparamagnetic iron oxide

With fluorescence microscopy (Fig. 4, 5) immunohistochemistry and immunofluorescence (Fig. 5), high numbers of AT-MSCs expressing GFP antigen were detected at and near the site of injection 3, 5, 7 and 9 weeks after intralesional injection in all horses. At visual inspection, there was a tendency of finding less labelled AT-MSCs with increasing time after implantation in sequential samples taken from different horses, respectively (Fig. 4). SPIO particles incorporated into AT-MSCs were detected by use of Prussian blue staining in horses 1, 2 and 4 (Fig. 6).

### MRI tracking of SPIO-labelled AT-MSCs

**Fig. 7:**
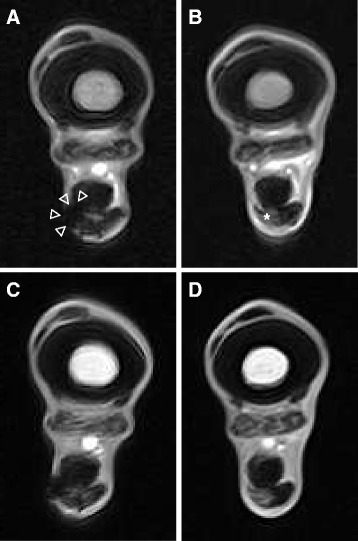
Transverse magnetic resonance imaging images of SDFT lesions treated with 10 × 10^6^ SPIO-labelled AT-MSCs or saline. **a, c** A focal, moderately demarcated hypointense area is evident in and lateral to the AT-MSC-treated SDFT lesion, area indicated by arrowheads in **a**. **b, d** Signal intensity of the saline treated lesion is increased in the contralateral SDFT, area indicated by asterisk in **b**. **a, b:** T2*-weighted sequence, 47 days after injection. **c**, **d**: T1-weighted sequence, 62 days after injection; horse 4. *AT-MSC* adipose tissue-derived mesenchymal stromal cell, *SDFT* superficial digital flexor tendon, *SPIO* superparamagnetic iron oxide

In horse 4, areas with hypointense signal, representing a magnetic susceptibility artefact resulting from SPIO particles, surrounded by regions of increased signal intensity, were detectable on transverse T2*-weighted MRI images in and around the SDFT at days 2, 18, 47 and 62 after treatment (Fig. 7a). T1-weighted images also showed a negative contrast in the lesions and in their lateral periphery (Fig. 7c). Areas of signal void corresponded to the two sites of injection in both sequences. In horse 2, a corresponding finding was present at 6 and 33 days after treatment. As compared with the contralateral SDFT, areas of decreased signal intensity were diffusely defined and markedly extended to the lateral subcutis in T2* and T1 sequences (Fig. 7).

## Discussion

### Main findings

The results of the present study show the potential of standing low-field MRI using T2*- and T1-weighted sequences to track SPIO-labelled AT-MSCs *in vivo* over a period of at least 9 weeks. It is further shown that GFP-labelled AT-MSCs can be successfully detected in and near surgically induced equine SDFT lesions between 3 to 9 weeks after intralesional injection by using fluorescence microscopy, immunofluorescence, immunohistochemistry and Prussian blue staining.

### AT-MSCs versus MSCs from other sources

In a previous study, immunophenotyping of equine MSCs showed that MSCs from different sources vary in their expression of MSC-defining antigens [22], suggesting that a deliberate choice of the cell type is important for the treatment of tendinopathy. In the present study, AT-MSCs were used because comparative *in vitro* investigations on the potential suitability for the treatment of tendinopathy made obvious that AT-MSCs express extracellular matrix proteins and tendon markers at significant rates, suggesting an outstanding potential of adipose-derived MSCs for the treatment of tendinopathy [11].

Up to now, data about cellular homing of AT-MSCs were available for a period of merely 7 days after implantation of the cells, which had been labelled with quantum dots (nanocrystals) in a collagenase model of equine tendinopathy [12]. Results of the present study prove the traceability of AT-MSCs labelled with GFP near the injection site for up to at least 9 weeks (62 days). By contrast, in a different study, the number of equine BM-MSCs decreased significantly after 10 days. At the same time, equine ESCs were constantly traceable in high numbers until 90 days after injection by using a labelling technique similar to the one in the present study [10]. These findings demonstrate the existence of differences between MSCs from different tissue sources and emphasize the necessity to research the fate of different types of equine MSCs separately, dose-dependently and for a period of time longer than in the present study.

The number of cells injected in the present study was relatively high and reflects current practice in equine orthopaedics. In contrast to the high numbers of GFP-positive AT-MSCs after 9 weeks during the present investigation, consistent GFP expression of ovine BM-MSCs could be detected at day 7 but no longer at day 14 after injection of 0.5 and 1 × 10^6^ cells in a recent ovine experimental tendinopathy study [23].

### Relevance of finding

The tendency of finding fewer labelled AT-MSCs at visual inspection with increasing time after implantation in the sequential samples taken from different horses suggests a decrease of the cells over time. Nevertheless, high numbers of cells were still present in and near the lesion site for up to 9 weeks after treatment. It has been postulated that the beneficial effects of MSCs on tendon healing observed clinically [24] and experimentally [3] in studies may be the result of either *de novo* formation of tendon-like tissue (i.e., a true regenerative effect via engraftment and differentiation) or through modulatory paracrine actions of the cells by the production of cytokines and growth factors [25]. From the findings of the present study, it can be concluded that cells remain at the lesion site for a sufficiently long period after AT-MSC treatment of equine tendinopathy and that both pathways are possible. This conclusion further supports the clinical use of MSCs in horses with centrally located tendinopathy of the SDFT and may be an explanatory factor for the clinical observation that stem cell therapy results in a significantly lower number of re-injuries than other treatments for tendinopathy in the horse [6, 7, 26].

### Reliability of the techniques

The transfection rate of cultured MSCs with reporter genes expressing GFP was approximately 99 % in the cells used for injection, as shown by flow cytometric analysis during passage 0. Fluorescence microscopy during passage 1 and flow cytometric analysis during passage 2 showed that the percentage of transfected MSCs had a tendency to decrease during early passages. This finding agrees with previous observations and is potentially caused by transgene silencing caused by epigenetic downregulation of transgenes leading to mosaic expression patterns [27, 28]. However, lentiviral vectors encoding GFP have been shown to lack significant negative effects on survival and differentiation potential of MSCs *in vitro* [28]. The transfection of cultured MSCs with reporter genes expressing GFP was successful for tracking AT-MSCs up to 9 weeks after implantation, which proves the suitability of this technique for AT-MSCs implanted into tendon defects as previously described for BM-MSCs and ESCs [10]. The labelling of MSCs with GFP by viral transfection is an established method in experimental animal studies [23, 29, 30]. The detection of GFP under fluorescence microscopy might be impaired by natural fluorescence of cell metabolites and structural components such as collagen in tissue specimen [31]. This phenomenon referred to as “autofluorescence” did not impair interpretation in the present study significantly as verified by immunohistochemical staining and immunofluorescence of GFP-expressing cells using DAB and Alexa 546 and by counterstaining of cell nuclei with DAPI.

In the present study, SPIO-labelled AT-MSCs could be detected with MRI in the lesion and its lateral periphery up to 9 weeks after implantation. The presence of SPIO particles was proved successfully with Prussian blue staining of tendon tissue post-mortem. Labelling of MSCs with SPIO particles has been previously used to track MSCs successfully in laboratory rodents and sheep [23, 32, 33]; the procedure affects their viability to some extent but insignificantly, although their proliferative capacity may be decreased [16, 17, 23, 32]. In a recent equine study, SPIO-labelled BM-MSCs could be successfully detected in cadaveric SDFTs by using 1.5-Tesla MRI [16]. The present study shows that SPIO labelling is also a practicable technique to track AT-MSCs injected in surgically created tendon lesions with 0.27-Tesla MRI in live horses in T2*-weighted sequences with the aid of the susceptibility artefact. Detection of SPIO particles was also successful in T1-weighted sequences, accepting a less distinct demarcation of the intact tendon tissue. The use of ultra-small SPIO particles might have increased the contrast in T1-weighted sequences. Alternative sequences, potentially appropriate to monitor SPIO-particles (e.g., 3DT1), were not available for the standing low-field MRI used in the present study. Angulation of the SDFTs relative to the magnetic field, using the magic angle effect, would probably have further improved MRI of SPIO particles [33]. Vaguely demarcated hypointense areas in the subcutis at the injection site helped to identify the sites of injection, especially in T1-weighted sequences. These signal voids are a potential sign of migration of the injected MSCs or of reflux of the cell suspension during or after injection. Although no resistance of the syringe plunger was encountered during injection, alternative injection techniques should be considered to avoid reflux from tendon lesions.

The present study proves that co-labelling of equine AT-MSCs with a lentiviral copGFP promotor and SPIO particles is feasible. Whether the procedure affects the biological activity of the cells (and, if so, to what extent) remains uncertain [23].

Alternative techniques to track MSCs are labelling with nanocrystals, which are resistant to metabolic degradation and have few cytotoxic effects [34, 35], and with technetium-99 hexamethyl propylene amine oxime, which allows detection of cells by nuclear scintigraphy but only for 24 h after administration [36]. Both techniques have been used successfully in experimentally induced equine tendon lesions [12, 37].

### Cellulitis

Horses 2 and 4 developed mild cellulitis after surgical induction of the lesions, the former in the limb treated with SPIO-labelled AT-MSC, the latter in both limbs but predominantly in the control limb. This complication most likely can be attributed to the surgical procedure or the aftercare during the days after surgery (or both), as clinical signs started before injection of AT-MSCs. Metaphylactic treatment with antimicrobials might have decreased the risk. However, a deliberate choice was made not to do this as there is evidence that some antimicrobials have the potential to influence tendon metabolism: oxytetracycline showed *in vitro* inhibition of suspensory ligament myofibroblast contractility [38], and oral doxycycline administration leads to inhibition of matrix metalloproteinase-13 (MMP-13) in rat tendons [39]. Cellulitis was restricted to the subcutaneous area and there were no indications that it extended into the created core lesion. Hence, no direct effect on migration behaviour of the cells could be expected. Nor are there indications that the cellulitis has hampered the modalities used in the present study to track implanted cells in any way.

### Limitations

The present study’s crucial limitation was that the number of labelled cells was not counted but determined semi-quantitatively in post-mortem specimen. The fact that in the present study 10 × 10^6^ AT-MSCs were implanted but that Guest et al. [10] injected 1 × 10^6^ BM-MSCs or ESCs might have influenced the results. This holds true also for the use of different models of tendinopathy in the present compared with other studies [10, 12]. Only a small number of horses were included in the present pilot study, and a staggered approach was chosen to monitor the presence of AT-MSCs over time. Therefore, the potential impact of inter-individual variations (e.g., age, gender and local infection) on the outcome cannot be determined on the basis of the present results. Tissue from the opposite front-limb SDFT was not examined histologically in the present study, but Carvalho et al. [12] could not detect AT-MSCs in the contralateral SDFT 7 days after intralesional injection of the ipsilateral SDFT. Histological tracking of MSCs in the regional lymph node (*Lnn. axillares superficiales*) using the techniques presented here should be included in future studies to further characterize AT-MSC distribution after injection. Another limitation is that the signal intensity change was not quantified objectively on MRI images. The loss of signal intensity may be objectified and a threshold may be established.

## Conclusions

Conclusively, standing low-field MRI has the potential to track SPIO-labelled AT-MSCs in surgically induced SDFT lesions for up to 9 weeks *in vivo*, as demonstrated by the detection of large numbers of labelled cells with histology, immunofluorescence and immunohistochemistry in post-mortem specimen of the lesions. Injection techniques have to be chosen deliberately to avoid reflux of the cell substrate injected. In future dose-dependent controlled clinical trials, numbers of cells retrieved in the tendons should be quantified and horses should be monitored for a longer period of time.

## Abbreviations

AT-MSC: Adipose tissue-derived mesenchymal stromal cell
BM-MSC: Bone marrow-derived mesenchymal stromal cell
bwt: Body weight
DAB: Diaminobenzidine tetrachloride
DAPI: 4′,6-diamidino-2-phenylindole
ESC: Embryonic stem cell
FA: Flip angle
GFP: Green fluorescent protein
IV: Intravenously
MRI: Magnetic resonance imaging
MSC: Mesenchymal stromal cell
PBS: Phosphate-buffered saline
SDFT: superficial digital flexor tendon
SPIO: Superparamagnetic iron oxide
TE: Echo time
TR: Relaxation time

## References

[1] Smith R, Young N, Dudhia J, Kasashima Y, Clegg P, Goodship A (2009). Effectiveness of bone-marrow-derived mesenchymal progenitor cells for naturally occurring tendinopathy in the horse. Regen Med.

[2] Nixon AJ, Dahlgren LA, Haupt JL, Yeager AE, Ward DL (2008). Effect of adipose-derived nucleated cell fractions on tendon repair in horses with collagenase-induced tendinitis. Am J Vet Res.

[3] Carvalho A de M, Badial PR, Álvarez LE, Yamada AL, Borges AS, Deffune E, et al. Equine tendonitis therapy using mesenchymal stem cells and platelet concentrates: a randomized controlled trial. Stem Cell Res Ther. 2013;4:85. 10.1186/scrt236 PMC3854756 23876512

[4] Conze P, van Schie HT, Weeren RV, Staszyk C, Conrad S, Skutella T (2014). Effect of autologous adipose tissue-derived mesenchymal stem cells on neovascularization of artificial equine tendon lesions. Regen Med.

[5] Carvalho AD, Alves ALG, de Oliveira PGG, Alvarez LEC, Amorim RL, Hussni CA (2011). Use of Adipose Tissue-Derived Mesenchymal Stem Cells for Experimental Tendinitis Therapy in Equines. J Equine Vet Sci.

[6] Leppänen M, Heikkilä P, Katiskalahti T, Tulamo RM. Follow up of recovery of equine tendon & ligament injuries 18–24 months after treatment with enriched autologous adipose-derived mesenchymal stem cells: a clinical study. Regen Med. 2009;4:Suppl. 2. S21.

[7] Dahlgren LA. Fat derived mesenchymal stem cells for equine tendon repair. Regen Med. 2009;4:Suppl. 2. S14.

[8] Prockop DJ, Kota DJ, Bazhanov N, Reger RL (2010). Evolving paradigms for repair of tissues by adult stem/progenitor cells (MSCs). J Cell Mol Med.

[9] Guest DJ, Smith MR, Allen WR (2008). Monitoring the fate of autologous and allogeneic mesenchymal progenitor cells injected into the superficial digital flexor tendon of horses: preliminary study. Equine Vet J.

[10] Guest DJ, Smith MR, Allen WR (2010). Equine embryonic stem-like cells and mesenchymal stromal cells have different survival rates and migration patterns following their injection into damaged superficial digital flexor tendon. Equine Vet J.

[11] Burk J, Gittel C, Heller S, Pfeiffer B, Paebst F, Ahrberg AB (2014). Gene expression of tendon markers in mesenchymal stromal cells derived from different sources. BMC Res Notes.

[12] Carvalho AM, Yamada AL, Golim MA, Alvarez LE, Hussni CA, Alves AL (2014). Evaluation of mesenchymal stem cell migration after equine tendonitis therapy. Equine Vet J.

[13] Wang YX, Hussain SM, Krestin GP (2001). Superparamagnetic iron oxide contrast agents: physicochemical characteristics and applications in MR imaging. Eur Radiol.

[14] Julke H, Veit C, Ribitsch I, Brehm W, Ludewig E, Delling U (2015). Comparative Labeling of Equine and Ovine Multipotent Stromal Cells With Superparamagnetic Iron Oxide Particles for Magnetic Resonance Imaging In Vitro. Cell Transplant.

[15] Wang HH, Wang YXJ, Leung KCF, Au DWT, Xuan SH, Chak CP (2009). Durable Mesenchymal Stem Cell Labelling by Using Polyhedral Superparamagnetic Iron Oxide Nanoparticles. Chem-Eur J.

[16] Bourzac CA, Koenig JB, Link KA, Nykamp SG, Koch TG (2014). Evaluation of ultrasmall superparamagnetic iron oxide contrast agent labeling of equine cord blood and bone marrow mesenchymal stromal cells. Am J Vet Res.

[17] Farrell E, Wielopolski P, Pavljasevic P, van Tiel S, Jahr H, Verhaar J (2008). Effects of iron oxide incorporation for long term cell tracking on MSC differentiation in vitro and in vivo. Biochem Biophys Res Commun.

[18] Li YG, Wei JN, Lu J, Wu XT, Teng GJ (2011). Labeling and tracing of bone marrow mesenchymal stem cells for tendon-to-bone tunnel healing. Knee Surg Sports Traumatol Arthrosc.

[19] Little D, Schramme MC (2006). Ultrasonographic and MRI evaluation of a novel tendonitis model in the horse. Vet Surg.

[20] Schramme M, Hunter S, Campbell N, Blikslager A, Smith R (2010). A surgical tendonitis model in horses: Techinque, clinical, ultrasonographic and histological characterisation. Vet Comp Orthopaed.

[21] Bosch G, van Schie HT, de Groot MW, Cadby JA, van de Lest CH, Barneveld A (2010). Effects of platelet-rich plasma on the quality of repair of mechanically induced core lesions in equine superficial digital flexor tendons: a placebo-controlled experimental study. J Orthop Res.

[22] Paebst F, Piehler D, Brehm W, Heller S, Schroeck C, Tarnok A (2014). Comparative Immunophenotyping of Equine Multipotent Mesenchymal Stromal Cells: An Approach Toward a Standardized Definition. Cytom Part A.

[23] Scharf A, Holmes S, Thoresen M, Mumaw J, Stumpf A, Peroni J (2015). Superparamagnetic iron oxide nanoparticles as a means to track mesenchymal stem cells in a large animal model of tendon injury. Contrast Media Mol Imaging.

[24] Smith RK, Werling NJ, Dakin SG, Alam R, Goodship AE, Dudhia J (2013). Beneficial effects of autologous bone marrow-derived mesenchymal stem cells in naturally occurring tendinopathy. PLoS One.

[25] Caplan AI (2009). Why are MSCs therapeutic? New data: new insight. J Pathol.

[26] Godwin EE, Young NJ, Dudhia J, Beamish IC, Smith RK (2012). Implantation of bone marrow-derived mesenchymal stem cells demonstrates improved outcome in horses with overstrain injury of the superficial digital flexor tendon. Equine Vet J.

[27] Kaufman WL, Kocman I, Agrawal V, Rahn HP, Besser D, Gossen M (2008). Homogeneity and persistence of transgene expression by omitting antibiotic selection in cell line isolation. Nucleic Acids Res.

[28] McGinley L, McMahon J, Strappe P, Barry F, Murphy M, O’Toole D (2011). Lentiviral vector mediated modification of mesenchymal stem cells & enhanced survival in an in vitro model of ischaemia. Stem Cell Res Ther.

[29] Czernik M, Fidanza A, Sardi M, Galli C, Brunetti D, Malatesta D (2013). Differentiation potential and GFP labeling of sheep bone marrow-derived mesenchymal stem cells. J Cell Biochem.

[30] Colosimo A, Curini V, Russo V, Mauro A, Bernabo N, Marchisio M (2013). Characterization, GFP gene Nucleofection, and allotransplantation in injured tendons of ovine amniotic fluid-derived stem cells. Cell Transplant.

[31] Lin CS, Xin ZC, Dai J, Lue TF (2013). Commonly used mesenchymal stem cell markers and tracking labels: Limitations and challenges. Histol Histopathol.

[32] Wang L, Deng JX, Wang J, Xiang B, Yang TH, Gruwel M (2009). Superparamagnetic iron oxide does not affect the viability and function of adipose-derived stem cells, and superparamagnetic iron oxide-enhanced magnetic resonance imaging identifies viable cells. Magn Reson Imaging.

[33] Yang Y, Zhang J, Qian Y, Dong S, Huang H, Boada FE (2013). Superparamagnetic iron oxide is suitable to label tendon stem cells and track them in vivo with MR imaging. Ann Biomed Eng.

[34] Collins MC, Gunst PR, Cascio WE, Kypson AP, Muller-Borer BJ (2012). Labeling and imaging mesenchymal stem cells with quantum dots. Methods Mol Biol.

[35] Muller-Borer BJ, Collins MC, Gunst PR, Cascio WE, Kypson AP (2007). Quantum dot labeling of mesenchymal stem cells. J Nanobiotechnol.

[36] Sole A, Spriet M, Galuppo LD, Padgett KA, Borjesson DL, Wisner ER (2012). Scintigraphic evaluation of intra-arterial and intravenous regional limb perfusion of allogeneic bone marrow-derived mesenchymal stem cells in the normal equine distal limb using (99 m) Tc-HMPAO. Equine Vet J.

[37] Sole A, Spriet M, Padgett KA, Vaughan B, Galuppo LD, Borjesson DL (2013). Distribution and persistence of technetium-99 hexamethyl propylene amine oxime-labelled bone marrow-derived mesenchymal stem cells in experimentally induced tendon lesions after intratendinous injection and regional perfusion of the equine distal limb. Equine Vet J.

[38] Arnoczky SP, Lavagnino M, Gardner KL, Tian T, Vaupel ZM, Stick JA (2004). In vitro effects of oxytetracycline on matrix metalloproteinase-1 mRNA expression and on collagen gel contraction by cultured myofibroblasts obtained from the accessory ligament of foals. Am J Vet Res.

[39] Bedi A, Fox AJ, Kovacevic D, Deng XH, Warren RF, Rodeo SA (2010). Doxycycline-mediated inhibition of matrix metalloproteinases improves healing after rotator cuff repair. Am J Sports Med.

